# A Novel Prototype Neonatal Resuscitator That Controls Tidal Volume and Ventilation Rate: A Comparative Study of Mask Ventilation in a Newborn Manikin

**DOI:** 10.3389/fped.2016.00129

**Published:** 2016-11-28

**Authors:** Anne Lee Solevåg, Enrico Haemmerle, Sylvia van Os, Katinka P. Bach, Po-Yin Cheung, Georg M. Schmölzer

**Affiliations:** ^1^Neonatal Research Unit, Centre for the Studies of Asphyxia and Resuscitation, Royal Alexandra Hospital, Edmonton, AB, Canada; ^2^Department of Pediatrics, University of Alberta, Edmonton, AB, Canada; ^3^Department of Pediatric and Adolescent Medicine, Akershus University Hospital, Lørenskog, Norway; ^4^School of Engineering, Computer and Mathematical Sciences, Auckland University of Technology, Auckland, New Zealand; ^5^Newborn Services, Auckland City Hospital, Auckland, New Zealand

**Keywords:** newborn, critical care, ventilation, bronchopulmonary dysplasia, tidal volume, resuscitation

## Abstract

The objective of this randomized controlled manikin trial was to examine tidal volume (V_T_) delivery and ventilation rate during mask positive pressure ventilation (PPV) with five different devices, including a volume-controlled prototype Next Step™ device for neonatal resuscitation. We hypothesized that V_T_ and rate would be closest to target with the Next Step™. Twenty-five Neonatal Resuscitation Program providers provided mask PPV to a newborn manikin (simulated weight 1 kg) in a randomized order with a self-inflating bag (SIB), a disposable T-piece, a non-disposable T-piece, a stand-alone resuscitation system T-piece, and the Next Step™. All T-pieces used a peak inflation pressure of 20 cmH_2_O and a positive end-expiratory pressure of 5 cmH_2_O. The participants were instructed to deliver a 5 mL/kg V_T_ (rate 40–60/min) for 1 min with each device and each of three test lungs with increasing compliance of 0.5, 1.0, and 2.0 mL/cmH_2_O. V_T_ and ventilation rate were compared between devices and compliance levels (linear mixed model). All devices, except the Next Step™ delivered a too high V_T_, up to sixfold the target at the 2.0-mL/cmH_2_O compliance. The Next Step™ V_T_ was 26% lower than the target in the low compliance. The ventilation rate was within target with the Next Step™ and SIB, and slightly lower with the T-pieces. In conclusion, routinely used newborn resuscitators over delivered V_T_, whereas the Next Step™ under delivered in the low compliant test lung. The SIB had higher V_T_ and rate than the T-pieces. More research is needed on volume-controlled delivery room ventilation.

## Introduction

Inappropriate tidal volume (V_T_) delivery during positive pressure ventilation (PPV) has been associated with an increased risk of both brain and lung injury in preterm infants (1, 2). Thus, modern mechanical ventilators use flow sensors to deliver volume-targeted ventilation. Perinatal transition involves rapidly changing lung mechanics (3), and despite the increasing focus on volume-targeted ventilation, commonly used neonatal resuscitation devices (4, 5) [e.g., self-inflating bags (SIBs) or T-piece devices] are pressure limited. If PPV is required, the inflation pressure and inflation time should be adjusted to deliver an adequate V_T_ as the lungs are gradually aerating and clearing lung liquid (6–8). However, complicating the task of providing PPV in the delivery room (DR) is the fact that the “appropriate” V_T_ varies in the first minutes after birth (9, 10). We have previously demonstrated that T-piece and SIB V_T_ were above the target in an intubated model with high airway compliance when a set peak inflation pressure (PIP) was used during PPV (11). However, mask ventilation differs from ventilation using an endotracheal tube (ETT) in many aspects. Distension of the facemask and upper airways during PPV might contribute to a difference in V_T_ needed to achieve adequate lung inflation during mask PPV compared to ETT ventilation (12). Mask leak and airway obstruction are other significant complicating factors during mask PPV (13, 14).

Thus, the primary aim of this study was to compare the same commonly used neonatal resuscitators that we used in our previous ETT study (11), to assess V_T_ delivery and ventilation rate during mask PPV with changing airway compliance in a newborn manikin. Also, similar to our previous study, we added a prototype ventilation device designed to deliver a predetermined V_T_ and ventilation rate irrespective of airway compliance (Next Step™, KM Medical, Auckland, New Zealand) for comparison. We hypothesized that the Next Step™ V_T_ delivery and ventilation rate would be most consistent compared to all other devices.

## Materials and Methods

### Environment and Subjects

The study was carried out in July 2015 at The Royal Alexandra Hospital, Edmonton, a tertiary perinatal center with an annual delivery rate of >7,000, and admitting approximately 1,500 infants to the Neonatal Intensive Care Unit (NICU) each year. The Northern Alberta Neonatal Program Research Committee and Health Ethics Research Board, University of Alberta approved the study. Registered Neonatal Resuscitation Program (NRP) health-care professionals including neonatologists, neonatal fellows, neonatal nurse practitioners, respiratory therapists, and neonatal nurses were included after written informed consent. The most commonly used neonatal resuscitator used by the participants was either T-piece device tested in the present study. The SIB was infrequently used.

### Randomization

This was a randomized, prospective, experimental study. All participants performed PPV with five ventilation devices in a randomized order. The primary investigator (Anne Lee Solevåg) conducted the randomization using an online tool^1^ and a code list. As this was a manikin study, the trial was not registered in the clinical trials database.^2^

### Sample Size Estimation

Our primary outcome was V_T_ delivery. A sample size of 25 NRP providers would be sufficient to detect a 40% more accurate V_T_ delivery with the Next Step™ device compared to a pressure-limited device, which we considered clinically important with 80% power and a two-tailed alpha error of 0.05.

### Manikin

**Figure 1:**
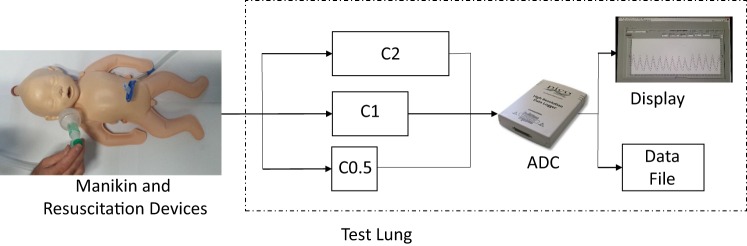
**Test lung components**. The upper airway of the manikin was connected *via* rubber tubing to three external test lungs. The cylinder test lungs were designed to exhibit a compliance of 0.5 mL/cmH_2_O (C0.5), 1.0 mL/cmH_2_O (C1), and 2.0 mL/cmH_2_O (C2), respectively. ADC, analog-to-digital converter.

We used a NeoNatalie manikin (Laerdal Medical, Stavanger, Norway) modified with rubber tubing connecting the manikin’s pharynx with test lungs with three different compliance levels (Figure 1). The manikin did not exhibit chest rise, and its exterior resembled a term infant.

### Test Lungs and Integrated Flow Sensor

Details about the three aluminum cylinder test lungs with integrated flow sensor are presented in Ref. (11). In agreement with clinical data (15), the test lungs had compliances of (i) 0.5 mL/cmH_2_O, (ii) 1.0 mL/cmH_2_O, and (iii) 2.0 mL/cmH_2_O, respectively. The test lungs were kept in a case and connected to the upper airways of the manikin by rubber tubing (Figure 1). The test lung settings were masked to participants during the study. Prior to each study day, the test lungs were checked for accuracy using a 10-mL glass syringe.

### Ventilation Devices and Mask

**Figure 2:**
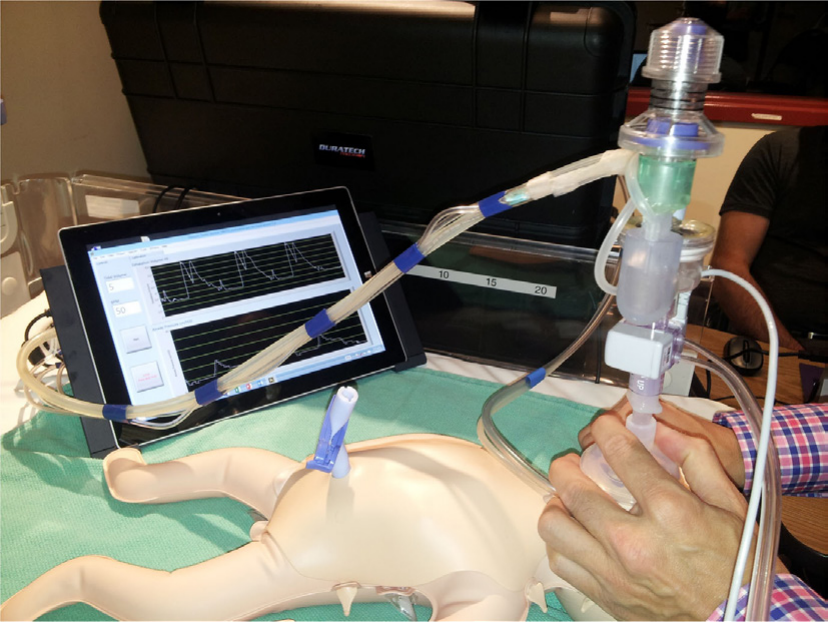
**Current prototype of the KM Medical Next Step™**.

Prior to the experiments, the participants had time to familiarize themselves with each ventilation device. (1) A SIB with a 35-cmH_2_O pop-off valve (Laerdal Medical, Stavanger, Norway), no PEEP valve, or manometer attached; (2) Neo-Tee disposable T-piece (Mercury Medical, Clearwater, FL, USA) with PIP 20 cmH_2_O and PEEP 5 cmH_2_O; (3) Neopuff™ Infant T-piece (Fisher & Paykel, Auckland, New Zealand) with PIP 20 cmH_2_O and PEEP 5 cmH_2_O; (4) Giraffe Stand-alone Infant Resuscitation System T-piece (GE Healthcare, Buckinghamshire, UK) with PIP 20 cmH_2_O and PEEP 5 cmH_2_O; and (5) The Next Step™ (KM Medical, Auckland, New Zealand) with a 5-cmH_2_O PEEP valve. The Next Step™ neonatal resuscitator (Figure 2) controls V_T_ and ventilation rate and monitors airway pressure. The 800g prototype is operated *via* a tablet displaying PIP, PEEP, and V_T_ in real time. According to the manufacturer, the device delivers V_T_ with an accuracy of 0.1–0.3 mL and has an internal battery with a life of ≥4 h in the standard configuration. For all devices, a Neonatal Clear Anatomical Face Mask (Mercury Medical) was used.

### Experimental Protocol

Each participant used all five ventilation devices in a randomized order, delivering PPV with each device for 3 × 1 min (1 min for each compliance level) targeting a rate of 40–60/min. The Next Step™ ventilation rate was set at 50/min. The 1-min ventilation sessions were separated by a minute during which compliance and/or ventilation device was changed and the participants were allowed to recover. The compliance levels were used in a predetermined order (0.5, 1.0, and 2.0 mL/cmH_2_O) for each device to simulate the changing airway compliance after birth (15, 16). Although participants knew that the compliance would change after each minute of PPV, they were unaware of the starting compliance level. Since small preterm infants are most prone to ventilation-induced lung injury (17), the experimental setup was that of a 1-kg infant and a target V_T_ of 5 mL/kg (18). During the experiments, participants were reminded to provide a targeted V_T_ of 5 mL/kg, but were blinded to respiratory data recordings, except when using the Next Step™, which has a screen displaying airway pressures and V_T_ in real time. After completion of all experiments, each participant was asked to complete a brief questionnaire. Questions included (i) “difficulty of providing PPV with each device”; (ii) “comfort with each device to provide PPV”; and (iii) preferred ventilation device; as well as profession, years of experience, and gender. Questions (i) and (ii) were rated using a Likert scale [1 (very difficult/uncomfortable) to 5 (very easy/comfortable)].

### Data Processing and Statistical Analyses

Demographical data and device preferences are presented as numbers and percentages or median with range or interquartile range (IQR). The primary outcome parameters of the study (V_T_ and ventilation rate with each device and compliance level) were tested for normality and presented as mean with 95% confidence interval. The outcome parameters were nested within participants, and a cluster effect was likely to be present in the data. As observations within the same participant tended to be correlated, differences between ventilation devices were estimated using a linear mixed model treating device and compliance as fixed effects and participants as random effects. Adjustments were made for multiple comparisons. Categorical data were compared between devices with the Pearson’s Chi-squared test.

Statistical analyses were performed in IBM SPSS Statistics 22 (IBM Corporation, Armonk, NY, USA). A *p*-value <0.05 was considered significant.

## Results

Twenty-five NRP providers were recruited for the study [neonatologists (*n* = 4), neonatal fellows (*n* = 5), neonatal nurse practitioners (*n* = 4), respiratory therapists (*n* = 8), and neonatal nurses (*n* = 4)]. Nineteen (76%) of the participants were female. Participants had a median (range) of 6 (1–26) years of neonatal experience, and 10 (0–22) months had passed since their last NRP update.

### Tidal Volume Delivery

The Next Step™ delivered the most consistent V_T_ at all compliance levels, but the V_T_ was 26% lower than the target at the lowest compliance level (0.5 mL/cmH_2_O). The SIB delivered a higher V_T_ than all other devices at all compliance levels. V_T_ delivery was similar between T-piece devices in all three compliance levels. In summary, all ventilation devices except the Next Step™ delivered V_T_, which was twofold to sixfold higher than our target V_T_ with increasing compliance levels (Table 1).

**Table 1:** **Tidal volumes during 1 min of positive pressure ventilation with different ventilation devices and compliance levels**.

Compliance	V_T_ (mL)			Rate (per minute)		
	0.5 mL/cmH_2_O (*n* = 25)	1.0 mL/cmH_2_O (*n* = 25)	2.0 mL/cmH_2_O (*n* = 25)	0.5 mL/cmH_2_O (*n* = 25)	1.0 mL/cmH_2_O (*n* = 25)	2.0 mL/cmH_2_O (*n* = 25)
SIB	11.4 (8.9–13.9)**	17.6 (13.2–22.0)**	23.5 (17.9–29.0)**	43 (38–49)**	42 (37–48)**	44 (38–50)**
Neo-Tee	5.6 (4.4–6.7)*	11.2 (9.0–13.3)*	19.3 (15.4–23.1)*	39 (32–46)**	38 (29–47)**	39 (32–46)**
Neopuff	6.1 (5.2–7.0)**	10.0 (7.4–12.5)**	21.3 (18.5–24.0)**	32 (26–38)**	40 (34–45)**	37 (32–42)**
Giraffe	5.7 (4.5–6.9)**	10.9 (8.5–13.3)**	19.8 (16.3–23.3)**	39 (35–43)**	39 (33–45)**	39 (33–44)**
Next Step™	3.7 (3.2–4.2)	4.9 (4.5–5.3)	4.5 (3.8–5.1)	48 (47–49)	49 (49–50)	50 (49–50)

*Values are presented as mean with 95% confidence interval*.

*V_T_, tidal volume; SIB, self-inflating bag*.

*p-Value = 0.018 vs. the Next Step™.

**p-Value ≤0.001 vs. the Next Step™.

### Ventilation Rate

Insignificant variation in the Next Step™ ventilation rate was observed (Table 2). By the design of the study, the ventilation rate with the Next Step™ was significantly different from that of the other devices i.e., set at 50 inflations/min, whereas the rate for the other devices was operator dependent. For the other devices, the ventilation rate was within 40–60/min at all compliance levels only with the SIB. The T-piece ventilation rates were slightly below the target at all compliance levels.

**Table 2:** **Health-care professionals’ Likert scale rating of how difficult and comfortable [1 (very difficult/uncomfortable) to 5 (very easy/comfortable)] it was to use the different ventilation devices**.

	How difficult was ventilation with the device? (*n* = 25)	How comfortable was the use of the ventilation device? (*n* = 25)
Self-inflating bag	3 (2–4)*	3 (2–4)*
Neo-Tee disposable T-piece	4 (3–4)	4 (3–4)
Neopuff infant T-piece	4 (4–5)	4 (4–5)
Giraffe stand-alone T-piece	4 (4–5)	4 (4–5)
Next step	5 (4–5)^†^	4 (3–5)

*Values are presented as median with interquartile range (IQR)*.

*p < 0.05 vs. all the other devices.

*^†^p < 0.05 vs. all the other devices*.

Questionnaire results are presented in Table 2. The Next Step™ was rated the easiest and the SIB the most difficult device to use. There was a significant difference in the number of participants preferring each device: 10 (40%) participants preferred the Next Step™, 9 (36%) preferred the Neopuff T-piece, 4 (16%) preferred the Giraffe T-piece, 1 (4%) preferred the Neo-Tee, and 1 (4%) answered “any T-piece device” (*p* < 0.001). The main difference was between the Neo-Tee and the Next Step™ (*p* = 0.002), and the SIB and the Next Step™ (*p* < 0.001).

## Discussion

In this randomized, controlled study comparing V_T_ delivery during mask ventilation at different compliance levels, we found that V_T_ delivery with widely used neonatal resuscitators was twofold to sixfold higher than our targeted V_T_ at high airway compliance, whereas volume delivery was lower than targeted with a prototype volume-controlled resuscitator at the lowest compliance.

During mask ventilation, the proportion of the V_T_ that enters the gas exchanging regions of the lungs may be affected by some of the volume being retained in the oropharynx and upper trachea (12). van Vonderen et al. (12) found that equivalent inflation pressures (25 cmH_2_O) resulted in significantly higher V_Ti_ (11.1 vs. 5.8 mL/kg) and V_Te_ (8.3 vs. 4.9 mL/kg) during mask ventilation in 10 preterm infants compared to ETT ventilation in the same infants after intubation. We have previously studied ETT ventilation in our manikin model (11), and when we compare the ETT results with mask data from the present study, we also find higher V_T_ delivery at the low and high compliance setting using a facemask compared to ETT. However, with a compliance of 1 mL/cmH_2_O, there is no difference in V_T_ delivery using a facemask or ETT with either device in our model.

### T-Piece Resuscitators

The participants in the current study were not allowed to adjust the PIP on the T-piece devices, which potentially could have yielded even higher V_T_ than measured. On the other hand, a decrease in PIP with increasing compliance might have resulted in lower V_T_ delivery. Although compliance changes during lung aeration at birth might be detected during PPV using a flow-inflating bag (19), it remains challenging using either a SIB or T-piece device (20–22). In fact, our results support this claim as we observed increasing V_T_ with increasing compliance levels (Table 1), despite the fact that the T-pieces are the devices most extensively used by the study participants. This is further supported by Huynh et al. (23), who reported that participants are unable to assess effectiveness of V_T_ delivery during compliance changes.

T-piece resuscitators have several advantages compared to a SIB, including more consistent V_T_ delivery (24, 25) and airway pressure (5, 10, 26–28), which was also observed in the current study and our previous ETT study (11).

### Self-Inflating Bag

The fact that SIBs are readily available in our NICU but rarely used might partly explain the fact that the participants found the SIB relatively difficult and uncomfortable to use during mask PPV (Table 2). A T-piece requires a gas source resulting in pressurizing the facemask, which could cause higher mask leak and lower V_T_ compared to the SIB. We did not measure mask leak, but this might have contributed to the higher V_T_ delivery with the SIB. However, the general lack of experience with the SIB might also have introduced a bias toward a greater deviation from target V_T_ with this device in our participants.

### The Next Step™

The prototype volume-controlled resuscitator used in this experiment utilizes room air and does not require a pressurized gas source. It can deliver supplementary oxygen if connected to a gas tank or oxygen outlet. Similar to our findings in an intubated manikin (11), the Next Step™ provided the most consistent V_T_ of all devices in the current study using a facemask (Table 1). Participants mentioned that the mask hold with the Next Step™ was unfamiliar, which might have resulted in a higher mask leak and V_T_ below target in the low compliance setting, especially since the low compliance was the first compliance tested. This suggests that health-care personnel should be familiar with the ventilation device they commonly use. However, despite this, the Next Step™ was ranked number one for preferred device as well as easiest to be used (Table 2). These are very important attributes, as the DR is often a stressful environment, where decisions are made quickly and resuscitators need to have good mask ventilation skills. In addition, the Next Step™ provides visual feedback by displaying delivered airway pressures and V_T_ during PPV. Although respiratory function monitoring is routinely used in the NICU (29), it is not commonly used in the DR during resuscitation (28, 30). Compared to a respiratory function monitor, the Next Step™ is expected to be cheaper. Also, being smaller and portable, the Next Step™ offers an advantage in units without a designated resuscitation area, i.e., where resuscitation is performed in the delivery suite. With these features, the Next Step™ device is a promising new device for neonatal resuscitation.

### Ventilation Rate

Even though the ventilation rate was close to the 40–60/min target with the SIB and T-piece devices, the 95% confidence intervals indicate a variable rate during manual PPV. Preterm infants may have impaired cerebral autoregulation (31), and changes in minute ventilation caused by inconsistent ventilation rate may result in blood CO_2_ fluctuations, which again have a theoretical potential for causing damage to the immature brain (32). Achieving more consistent ventilation rates in the DR offers a potential for improved outcomes.

### Limitations

Using a manikin to simulate DR resuscitation has its limitations, as it does not resemble real-life resuscitation. However, new ventilation devices have to undergo extensive bench-top testing prior to introduction into the DR. During real-life resuscitation, clinicians can use clinical cues (e.g., changes in heart rate) to adjust their ventilation efforts (33), which manikins do not have. Furthermore, the manikin resembled a term infant rather than a 1-kg preterm infant and chest rise was not visible as the manikin was connected to the external test lungs, which might have caused higher V_T_ delivery. We tried to reduce this bias by continuously reminding the participants that they should aim for a 5-mL V_T_/kg.

In conclusion, the Next Step™, a volume-controlled device, had the most consistent V_T_ delivery and ventilation rate compared to all other devices, and the SIB over delivered to the greatest extent. Participants were unable to recognize compliance changes. The Next Step™, a volume-targeted neonatal resuscitation device has the potential to provide a lung-protective strategy from birth both using a facemask and ETT, but requires further investigation.

## Author Contributions

AS conceptualized and designed the study; performed the data collection, data analysis, and data interpretation; and wrote the manuscript. EH, P-YC, and GS conceptualized and designed the study, was involved in the data analysis and data interpretation, critically reviewed the manuscript, and approved the final manuscript as submitted. SO and KB conceptualized and designed the study, was involved in the data interpretation, critically reviewed the manuscript, and approved the final manuscript as submitted.

## Conflict of Interest Statement

KM Medical provided The Next Step™ Neonatal Resuscitator for the study. The company was involved in the design of the study, but not in the data acquisition, data analysis, interpretation of the results, or writing of the manuscript. The test lung was designed and manufactured by Auckland University of Technology under the supervision of EH. Ms. SO and AS, KB, P-YC, and GS have no potential conflicts of interest relevant to this article to disclose. EH was in 2008 involved as a University of Auckland Senior Lecturer in the prototype design of the Next Step™ under a research and consultancy agreement between Auckland UniServices Limited and KM Medical (Auckland, New Zealand). Ms. SO and AS, KB, P-YC, and GS have no financial relationships relevant to this article to disclose.

## Footnotes

^1^http://www.randomizer.org.

^2^http://clinicaltrials.gov.
